# Low erythropoietin levels predict faster renal function decline in diabetic patients with anemia: a prospective cohort study

**DOI:** 10.1038/s41598-019-51207-8

**Published:** 2019-10-16

**Authors:** Yohei Fujita, Yohei Doi, Takayuki Hamano, Masahiro Hatazaki, Yutaka Umayahara, Yoshitaka Isaka, Yoshiharu Tsubakihara

**Affiliations:** 1 Department of Diabetes and Metabolism, Osaka General Medical Center, Osaka, Japan; 2 Department of Nephrology, Osaka University Graduate School of Medicine, Suita, Japan; 3 Department of Inter-Organ Communication Research in Kidney Disease, Osaka University Graduate School of Medicine, Suita, Japan; 4 Department of Nephrology, Nagoya City University Graduate School of Medical Sciences, Nagoya, Japan; 5 Graduate School of Health Care Sciences, Jikei Institute, Yodogawa-ku, Osaka, Japan

**Keywords:** Diabetes complications, Kidney diseases

## Abstract

Elevated erythropoietin (EPO) levels have been reported to predict poor survival in various populations including diabetic patients. However, data regarding its impact on renal outcomes are scarce. We conducted a single-center, prospective cohort study of 339 type 2 diabetic patients with anemia. The primary outcome was the estimated glomerular filtration rate (eGFR) slope for two years. We performed multiple linear regression and restricted cubic spline analyses to assess the association of serum EPO levels with the renal outcome. Chronic kidney disease (CKD) was defined as eGFR <60 mL/min/1.73 m2 or urine albumin-to-creatinine ratio >30 mg/g creatinine. Median baseline EPO and eGFR level were 14.4 IU/L and 53 mL/min/1.73 m^2^, respectively. Inappropriately low EPO levels were observed in 73% of anemic patients and 59% of anemic patients even without CKD, suggesting that EPO deficiency precedes the onset of CKD in diabetes mellitus. Multivariable analysis revealed that iron status and hemoglobin levels were major determinants of EPO levels. Median eGFR slope was −1.3 mL/min/1.73 m^2^/year. We found that low EPO levels, but not low hemoglobin levels, were associated with a faster decline in eGFR, independent of clinically relevant factors. The eGFR decline was steeper, particularly when the EPO level was below the upper limit of normal. Lower EPO concentrations were associated with rapid eGFR decline, especially in patients with iron deficiency (P for interaction = 0.01). Relative EPO deficiency should be considered as a culprit in anemia of unknown etiology in diabetic patients, even those without CKD. Low EPO levels, especially when accompanied by poor iron status, are predictive of rapid loss of renal function.

## Introduction

Diabetes mellitus is the leading cause of end-stage kidney disease (ESKD) and a risk factor for cardiovascular disease (CVD) and mortality^1^. Albuminuria, the earliest clinical manifestation of diabetic kidney disease (DKD), is a marker that predicts the progression of kidney disease and CVD^2,3^. The deterioration of glomerular filtration rate (GFR) was thought to occur in parallel with the onset of macroalbuminuria^4^. However, recent studies showed that an accelerated GFR decline precedes the incidence of albuminuria in patients with type 2 diabetes^4^. As little is known about the factors responsible for progressive GFR decline in such patients, an elucidation of predictive biomarkers other than albuminuria is important.

Anemia is common in diabetic patients and is associated with the major diabetic complications including nephropathy, retinopathy, and CVD^5^. Some studies demonstrated that low hemoglobin (Hgb) concentration is a risk factor for the progression of DKD, with or without albuminuria^6–8^. Although several factors contribute to the high prevalence of anemia in diabetes, erythropoietin (EPO) deficiency seems to be one of the major causes^5^. While it was reported that elevated EPO levels are associated with poor survival in heart failure, diabetic chronic kidney disease (CKD), and kidney transplant recipients^9–11^, its impact on renal outcomes remains to be determined. Therefore, we conducted a prospective cohort study in type 2 diabetic patients with anemia to assess the association of EPO levels with renal outcomes.

## Materials and Methods

### Study population

This study was a single-center, prospective observational study conducted at Osaka General Medical Center. In 2013, we screened type 2 diabetic patients in the outpatient Diabetes and Metabolism Department to identify those aged 20–85 years with anemia. The definition of anemia was a Hgb level <13.0 g/dL in men and <12.0 g/dL in women according to the World Health Organization criteria. We enrolled all subjects who gave informed consent, excluding dialysis patients and erythropoiesis-stimulating agent (ESA) users. The study was approved by the ethics committee of Osaka General Medical Center (Approval Number 27-C0418) and was conducted in accordance with the Helsinki Declaration. All participants provided written informed consent.

### Baseline characteristics and laboratory parameters

Previous CVD included a history of stroke, coronary artery disease, congestive heart failure, or peripheral arterial disease. Blood and urine samples were obtained at enrollment. After a 30-min incubation time, blood samples were centrifuged for serum separation and the sera were frozen and stored at −80 °C until the analyses. Chemistry parameters were measured using standard automated techniques. Serum EPO concentration was measured using a chemiluminescent enzyme immunoassay (Access EPO®; Beckman Coulter, Tokyo, Japan), with a normal range of 4.2–23.7 IU/L^12^. EPO levels below the upper normal limit (23.7 IU/L) were considered inappropriately low given the presence of anemia (relative EPO deficiency). The biologically active form of FGF23 (intact FGF23) was measured using a sandwich enzyme-linked immunosorbent assay (ELISA) system (Kainos Laboratories, Tokyo, Japan). Serum 25-hydroxyvitamin D (25D) was assessed by chemiluminescence immunoassay (Liaison 25-hydroxyvitamin D Total®; Kyowa Medex, Tokyo, Japan). Urinary liver-type fatty acid-binding protein (u-LFABP) was evaluated using a two-step sandwich ELISA system (CMIC Holdings, Tokyo, Japan). The estimated GFR (eGFR) was calculated according to a standard Japanese formula based on insulin clearance: 194 × creatinine^1.094^ × age^−0.287^ (if female × 0.739)^13^. Iron deficiency (ID) was defined as serum ferritin levels ≤50 ng/dL^14^. CKD was defined as eGFR <60 mL/min/1.73 m^2^ or urine albumin-to-creatinine ratio >30 mg/g creatinine.

### Outcome of interest

Patients were followed regularly at the outpatient clinic every two months until death or loss to follow-up. We defined the study outcome as the annualized eGFR slope estimated by ordinary least square regression fitting of all eGFR values in each subject for two years.

### Statistical analyses

Data are expressed as the median and interquartile range (IQR). Categorical variables are expressed as proportions. Differences between the EPO level categories were tested using the Wilcoxon rank-sum and chi-squared tests, as appropriate. Variables with skewed distribution were log transformed. For the baseline analyses, multivariable linear regression analyses with robust variances were performed to assess clinical and biochemical associations with log-transformed EPO levels as a dependent variable. Next, we employed linear regression analyses with robust variances to evaluate the association of baseline log-transformed EPO levels with eGFR slope. We hierarchically adjusted for the following confounders: age, sex, eGFR, urine albumin-to-creatinine ratio (UACR), and Hgb (model 1); model 1 plus diabetic retinopathy, systolic blood pressure (SBP), and hemoglobin A1c (HbA1c) (model 2); model 2 plus usage of angiotensin-converting enzyme inhibitor (ACEI) and/or angiotensin II receptor blocker (ARB) and statins (model 3); model 3 plus FGF23 and 25D (model 4); and model 4 plus u-LFABP (model 5). The covariates entered in the multivariable models were selected based on clinical knowledge and previous reports. As the association between EPO level and the eGFR slope may be non-linear, we also applied restricted cubic spline models with three knots. Interactions between age, sex, eGFR, C-reactive protein (CRP), Hgb, and ID and EPO level were examined, and stratified analyses were performed only when the interaction was significant. The statistical test was two-tailed, and P < 0.05 was considered statistically significant. All statistical analyses were performed using Stata/SE 15 (Stata Corp., College Station, TX).

## Results

### Baseline characteristics

**Figure 1:**
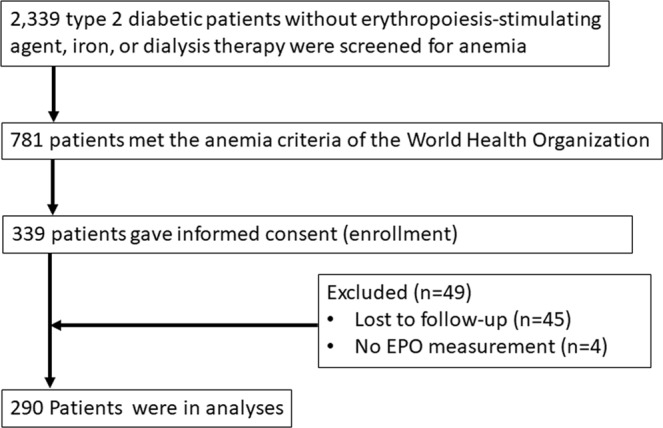
Flow chart of study population.

Of the 339 enrolled participants, we excluded 49 (14.5%) patients mainly due to lost to follow-up within two years. No patients died, initiated dialysis therapy, or received kidney transplantation during the study period. The final cohort included 290 patients (Fig. 1). No significant differences were observed in age, sex, eGFR, UACR, Hgb, HbA1c, or EPO levels between the included and excluded patients (data not shown). Less than 2% of our data were missing.

**Figure 2:**
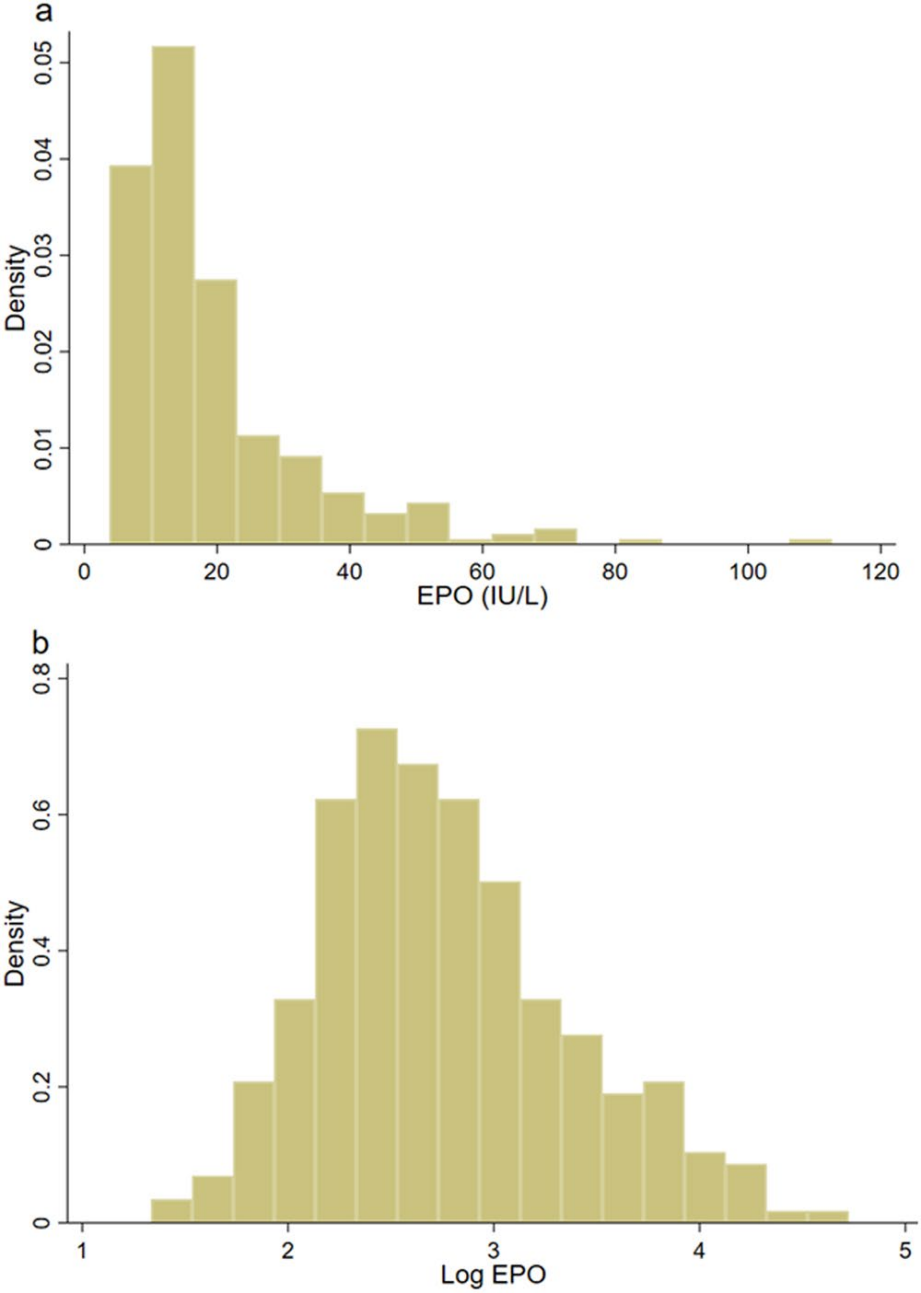
Histogram distribution of (**a**) EPO and (**b**) log-transformed EPO. Abbreviations: EPO, erythropoietin.

**Figure 3:**
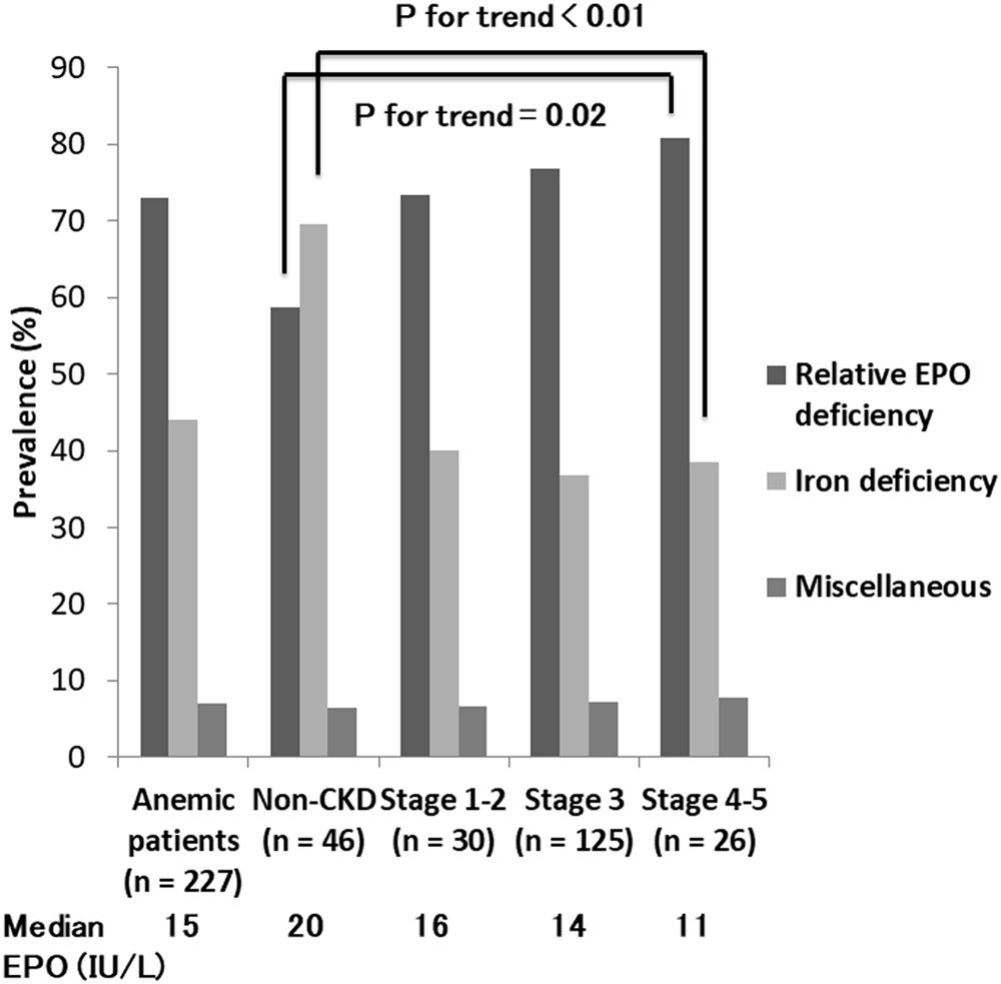
The prevalence of relative EPO deficiency and iron deficiency stratified by CKD stages in anemic patients. Of patients with informed consent, 227 (78%) patients met the anemia criteria again at the time of enrollment. Non-CKD and CKD stages were defined as follows: Non-CKD and CKD stages were defined as follows: non-CKD, eGFR ≥60 mL/min/1.73 m^2^ and UACR <30 mg/g, CKD stage 1–2, eGFR ≥60 mL/min/1.73 m^2^ and UACR ≥30 mg/g creatinine; stage 3, eGFR <60 and ≥30 mL/min/1.73 m^2^; stage 4–5, eGFR <30 mL/min/1.73 m^2^. Abbreviations: EPO, erythropoietin; CKD, chronic kidney disease.

Baseline characteristics are listed in Table 1, stratified by the median EPO level of 14.4 IU/L. At enrollment, 78% of the participants again met the anemia criteria. The median age was 71 years, and approximately 60% of the patients were men. The median eGFR and Hgb were 53 mL/min/1.73 m^2^ and 11.8 g/dL, respectively. The patients with macroalbuminuria (i.e., UACR ≥300 mg/gCr) accounted for 26.3% of the participants. Generally, HbA1c levels were well controlled. Overall, despite low Hgb levels, EPO levels were inappropriately low (median 14.4 IU/L). The distribution of EPO levels and log-transformed EPO is shown in Fig. 2. The low EPO group, defined as the group of patients with EPO levels less than 14.4 IU/L, was more likely to be younger and have diabetic retinopathy and lower BMI than the high EPO group. The low EPO group also showed higher Hgb, serum albumin, transferrin saturation (TSAT), and ferritin levels compared to the high EPO group. Figure 3 shows the prevalence of anemic patients with relative EPO deficiency and ID, which were 73.1% and 44.1%, respectively. Even in patients without CKD, i.e., eGFR ≥60 mL/min/1.73 m^2^ and UACR <30 mg/g creatinine, a substantial proportion of patients (58.7%) showed inappropriately low EPO levels. As the CKD stages progressed, the proportion of patients with relative EPO deficiency increased (P for trend = 0.02), whereas that of patients with ID decreased (P for trend <0.01). The median EPO levels fell parallelly with CKD progress (P for trend <0.01). Because the strict definition of CKD requires more than two measurements of eGFR and/or UACR at least 3 months apart, we reviewed eGFR and UACR more than 3 months before the enrollment. Finally, only 3 patients (1.3%) did not meet the strict CKD criteria. Re-classification of CKD based on the strict definition did not change the above-mentioned results substantially (data not shown).

**Table 1:** Patient characteristics categorized by the median EPO level.

Variables	All patients	Low EPO	High EPO	P value
n	290	145	145	
EPO, IU/L	14.4 (10.2–22.8)	10.2 (8.4–12.1)	22.8 (17.9–33.6)	<0.01
Age, years	71 (64–77)	70 (63–76)	72 (66–78)	0.04
Male sex	176 (61)	88 (61)	88 (61)	1.00
BMI, kg/m^2^	23.5 (21.1–26.8)	23.1 (20.7–25.8)	23.9 (21.5–27.8)	0.04
History of CVD	135 (47)	61 (42)	74 (51)	0.13
Diabetic retinopathy	119 (41)	68 (47)	51 (35)	0.04
Smoking	44 (15)	20 (14)	24 (17)	0.51
SBP, mmHg	130 (122–138)	129 (122–138)	130 (122–137)	0.73
Hemoglobin A1c, %	6.9 (6.3–7.4)	6.9 (6.4–7.4)	6.9 (6.3–7.5)	0.75
LDL-cho, mg/dL	95 (79–116)	98 (79–116)	95 (78–117)	0.82
eGFR, mL/min/1.73 m^2^	53 (40–67)	49 (40–63)	56 (40–71)	0.08
Corrected Ca, mg/dL	9.2 (9.0–9.4)	9.3 (9.0–9.4)	9.2 (9.0–9.4)	0.93
Albumin, g/dL	4.1 (3.9–4.4)	4.2 (3.9–4.4)	4.1 (3.8–4.3)	0.04
CRP, mg/dL	0.06 (0.01–0.15)	0.06 (0.01–0.11)	0.06 (0.01–0.22)	0.12
Hemoglobin, g/dL	11.8 (11.0–12.5)	11.9 (11.5–12.6)	11.4 (10.8–12.4)	<0.01
TSAT, %	23.4 (15.9–31.1)	25.9 (20.2–32.1)	19.7 (10.9–29.1)	<0.01
Ferritin, ng/dL	69 (26–214)	88 (46–141)	32 (14–103)	<0.01
UACR, mg/g Cre	60 (16–320)	74 (20–359)	47 (14–276)	0.11
u-LFABP, mg/g Cre	5.1 (2.3–14.2)	5.6 (2.5–15.7)	4.4 (2.1–11.8)	0.17
FGF23, pg/mL	32 (27–45)	34 (27–49)	33 (27–42)	0.31
25D, ng/mL	8 (5–13)	9 (5–13)	7 (5–14)	0.31
ACEI/ARB	177 (61.0)	93 (64.1)	84 (57.9)	0.28
Statin	113 (40)	61 (42)	52 (37)	0.34
Insulin	150 (53)	77 (54)	73 (52)	0.77
OHA	213 (74.7)	103 (71.0)	110 (75.9)	0.35
**Outcome**				
eGFR slope, mL/min/1.73 m^2^/year	−1.3 (−3.6–1.0)	−1.7 (−4.0–0.4)	−0.8 (−3.3–1.4)	0.02

Data are expressed as median (interquartile range) or n (%). EPO, erythropoietin; BMI, body mass index; CVD, cardiovascular disease; SBP, systolic blood pressure; eGFR, estimated glomerular filtration rate; Ca, calcium; CRP, C-reactive protein; TSAT, transferrin saturation; Cre, urine creatinine; UACR, urine albumin-to-creatinine ratio; u-LFABP, urinary liver-type fatty acid-binding protein; FGF23, fibroblast growth factor 23; 25D, 25-hydroxyvitamin D; ACEI/ARB, angiotensin-converting enzyme inhibitors or angiotensin receptor blockers; OHA, oral hypoglycemic agents.

### Clinical variables associated with EPO levels

For the baseline analyses, we performed multivariable linear regression analyses to assess the clinical and biochemical associations with EPO levels as a dependent variable (Table 2). Older age, higher BMI, eGFR, and CRP, and lower Hgb and ferritin were associated with higher EPO levels. Among these variables, Hgb and ferritin levels were strongly associated with EPO levels (standardized β coefficient −0.33 and −0.32, respectively).

**Table 2:** Multivariable linear regression analysis for log-transformed EPO.

Variable	Standardized β-coefficient	95% CI	P value
Age	0.14	0.03–0.24	0.01
Male sex	0.10	−0.01–0.22	0.07
BMI	0.16	0.04–0.29	0.01
eGFR	0.20	0.07–0.33	0.02
Log UACR	0.01	−0.14–0.17	0.87
Log u-LFABP	−0.02	−0.13–0.10	0.78
Hemoglobin	−0.33	−0.45–0.20	<0.01
HbA1c	−0.01	−0.13–0.11	0.85
Log ferritin	−0.32	−0.45–0.20	<0.01
Smoking	0.00	−0.09–0.09	0.93
Log CRP	0.12	0.01–0.23	0.04
Log FGF23	0.07	−0.06–0.19	0.30
ACEI/ARB	−0.07	−0.18–0.05	0.25
Previous CVD	0.03	−0.07–0.14	0.54

EPO, erythropoietin; CI, confidence interval; BMI, body mass index; eGFR, estimated glomerular filtration rate; UACR, urine albumin-to-creatinine ratio; u-LFABP, urinary liver-type fatty acid-binding protein; HbA1c: hemoglobin A1c; CRP, C-reactive protein; FGF23, fibroblast growth factor 23; ACE/ARB, angiotensin-converting enzyme inhibitors or angiotensin receptor blockers; CVD, cardiovascular disease.

### The relationship between EPO levels and the presence of anemia

In the whole cohort, an unadjusted logistic regression model showed that high EPO levels were associated with the presence of anemia (Odds ratio 1.32 per 1 SD increase in log-transformed EPO, 95% CI: 1.02–1.71). Since iron deficiency and inflammation (reflected by CRP) are well-known causes of anemia and these factors have positive relationships with EPO levels, we excluded patients with iron deficiency (i.e., ferritin levels ≤50 ng/dL) and elevated CRP (greater than the median value). After this exclusion, high EPO levels were not likely to be anemic (odds ratio 0.52 per 1 SD increase in log-transformed EPO, 95%CI 0.29–0.92).

### Outcome

**Figure 4:**
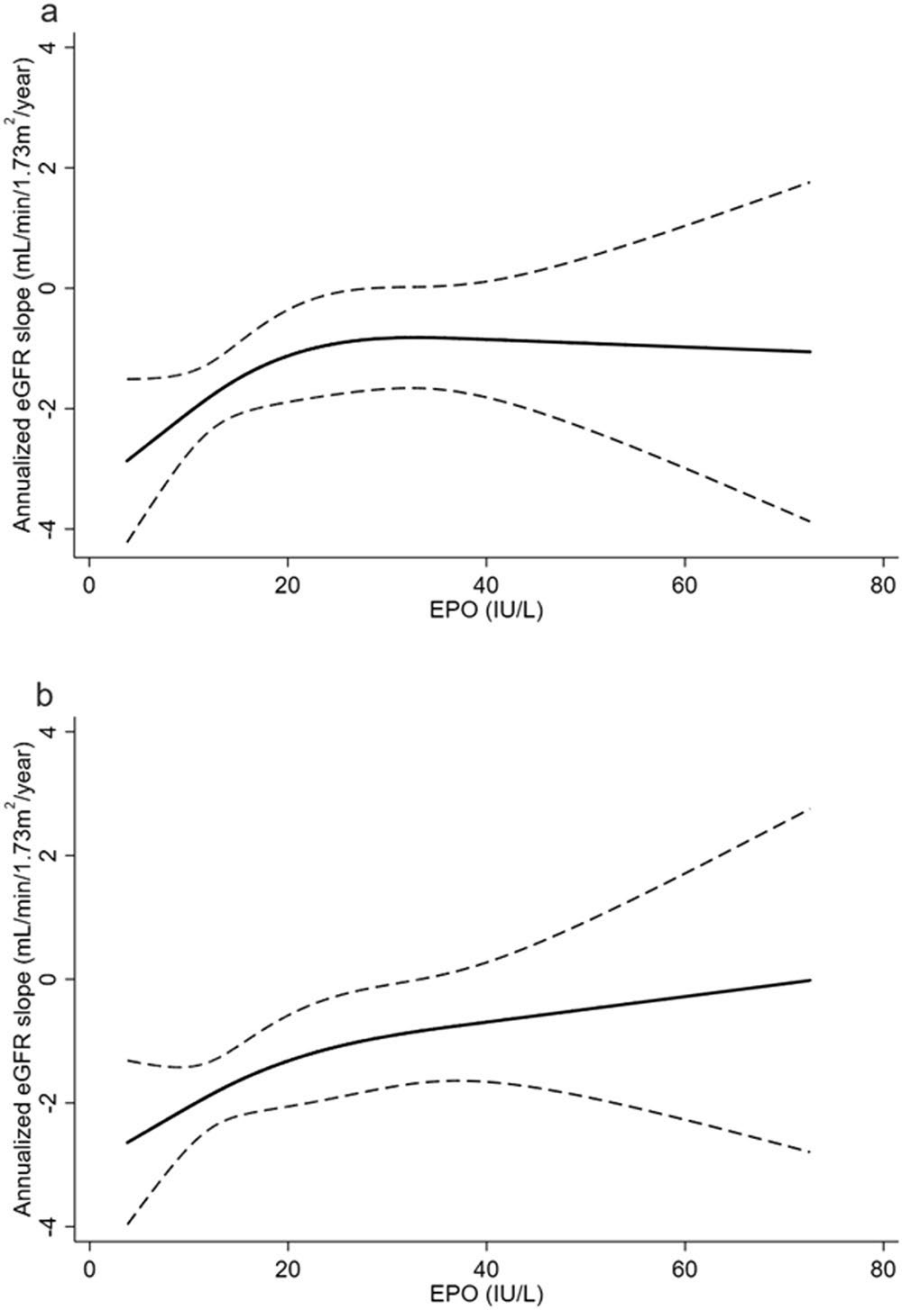
(**a**) Unadjusted and (**b**) adjusted associations of EPO levels with annualized eGFR slope. In the multivariable analysis, we adjusted for age, sex, eGFR, urine albumin-to-creatinine ratio, hemoglobin, diabetic retinopathy, systolic blood pressure, hemoglobin A1c, use of angiotensin-converting enzyme inhibitors or angiotensin receptors, statin use, fibroblast growth factor 23, 25-hydroxyvitamin D, and urinary liver-type fatty acid-binding protein. The solid line represents the point of estimate and the dashed line represents the 95% CI. Abbreviations: EPO, erythropoietin; eGFR, estimated glomerular filtration rate.

**Figure 5:**
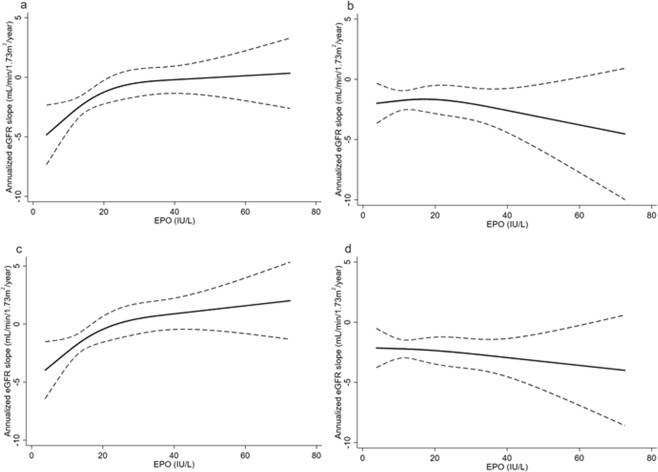
Association of EPO levels with annualized eGFR slope with (**a,c**) or without (**b,d**) iron deficiency. Iron deficiency was defined as ferritin ≤50 ng/mL in (**a,b**) or as TSAT ≤20% in (**c,d**). Models were adjusted for age, sex, eGFR, urine albumin-to-creatinine ratio, hemoglobin, diabetic retinopathy, systolic blood pressure, hemoglobin A1c, angiotensin-converting enzyme inhibitors or angiotensin receptor use, statin use, fibroblast growth factor 23, 25-hydroxyvitamin D, and urinary liver-type fatty acid-binding protein. The solid line represents the point of estimates and the dashed line represents the 95% CI. Abbreviations: EPO, erythropoietin; eGFR, estimated glomerular filtration rate.

The median eGFR slope was −1.3 mL/min/1.73 m^2^/year. The patients in the low EPO group had a significantly faster eGFR decline compared to those in the high EPO group (Table 1). Table 3 shows the association between baseline EPO levels and annualized eGFR slope using a linear regression model. In the crude model, baseline log-transformed EPO levels were significantly associated with eGFR decline (β coefficient 0.83, 95% CI: 0.03–1.64). This association remained significant even after full adjustment for clinically relevant factors (model 5: β coefficient 0.93, 95% CI: 0.05–1.81). In addition, the effect sizes of EPO levels on eGFR slope were consistent across models. While the relationship between log-transformed EPO levels and eGFR slope showed a linear relationship (P for linearity = 0.53), the relationship between EPO levels and eGFR slope proved to be non-linear (P for linearity = 0.02). Therefore, we applied restricted cubic spline models to represent the relationship between EPO levels and eGFR slope (Fig. 4). The eGFR slope was steeper in the crude and adjusted models, particularly below the upper normal limit (23.7 IU/L) of EPO level. Additionally, we assessed the effect modifications by age, sex, eGFR, CRP, Hgb, and ID by including interaction terms with these variables and EPO levels in model 5. We identified a significant interaction between log-transformed EPO levels and ID (P for interaction <0.01). We also confirmed similar results of interaction between EPO levels and ID in a non-linear relationship (P for interaction = 0.01). No interactions were found between EPO levels and the other variables (all P values for interaction >0.1). Therefore, after stratifying the patients into two groups with or without ID, we evaluated the association between EPO levels and eGFR slope in each group. Linear regression analyses showed that log-transformed EPO levels were significantly associated with eGFR slope in patients with ID (β coefficient 1.93, 95% CI 0.72–3.14), but not in those without ID (β coefficient −0.18, 95% CI −1.45–1.08) in model 5. Concordant results were observed when we redefined ID as TSAT ≤20% (β coefficient 2.2, 95% CI 0.90–3.54 in patients with ID and β coefficient −0.48, 95% CI −1.69–0.72 in patients without ID). We confirmed the effect modification by ID using restricted cubic spline analyses. Figure 5 shows that the slope changed from steep to gentle as EPO concentrations increased in the patients with ID (a, c), whereas no significant relationship was found in the patients without ID (b, d). In subgroup analyses excluding patients without anemia at enrollment (21.7%), the results remain substantially unchanged (data not shown).

**Table 3:** Linear regression analysis for eGFR slope.

Model	Variables	β-coefficient	95% CI	P value
crude	Log EPO	0.83	0.03–1.64	0.04
1	Crude + age, sex, eGFR, log UACR, and hemoglobin	0.86	0.01–1.71	0.05
2	Model 1 + diabetic retinopathy, SBP, and HbA1c	0.90	0.03–1.76	0.04
3	Model 2 + ACE/ARB use and statin use	0.89	0.01–1.77	0.05
4	Model 3 + log FGF23 and 25D	0.94	0.07–1.81	0.04
5	Model 4 + log u-LFABP	0.93	0.05–1.81	0.04

eGFR, estimated glomerular filtration rate; EPO, erythropoietin; CI, confidence interval; UACR, urine albumin-to-creatinine ratio; SBP, systolic blood pressure; HbA1c: hemoglobin A1c; ACE/ARB, angiotensin-converting enzyme inhibitors or angiotensin receptor blockers; FGF23, fibroblast growth factor 23; 25D, 25-hydroxyvitamin D; u-LFABP, urinary liver-type fatty acid-binding protein.

## Discussion

In the present study, we investigated the prognostic role of serum EPO levels in type 2 diabetic patients with anemia. The results showed that low EPO levels, particularly below the level of the upper normal limit (23.7 IU/L), predict rapid eGFR decline independently of established prognostic factors including eGFR, UACR, and Hgb. ID proved to be a significant interactive factor in the relationship between EPO and eGFR slope, as lower EPO concentrations were associated with rapid eGFR decline only in the patients with ID.

Previous studies revealed that EPO levels in diabetic patients are low compared with those in non-diabetic patients^15,16^. Several mechanisms have been proposed for the low EPO levels in diabetic patients, including abnormal anemia-sensing mechanisms owing to diabetic autonomic neuropathy, impaired production of EPO due to tubulointerstitial damage, and dysfunction of hypoxia inducible factor (HIF)^5^. In the current study, three-quarters of anemic patients showed inappropriately low EPO levels despite the presence of anemia. Surprisingly, we found that approximately 60% of patients without CKD had relative EPO deficiency. Although a number of studies have shown that EPO deficiency emerges early in DKD^15,16^, to our knowledge, no studies have reported EPO deficiency in diabetic patients without CKD. After excluding patients with anemia of known etiologies (i.e., ID and inflammation), low EPO levels were associated with the presence of anemia. EPO deficiency should be considered as a factor in anemia of unknown etiology in diabetic patients, even in those without CKD.

Furthermore, we demonstrated that Hgb and iron status were two major determinants of EPO levels in our study. EPO synthesis is regulated by HIF in renal EPO-producing cells (REPs)^17^. A drop in Hgb levels causes renal hypoxia, leading to the activation of EPO synthesis in patients with preserved renal function. Iron plays an essential role in oxygen delivery and is a critical cofactor for prolyl hydroxylase domain-containing enzymes (PHDs), which hydroxylate HIF^17^. The activity of PHDs depends not only on local oxygen pressure but also on iron availability^17^. We speculate that ID leads to HIF stabilization via inactivation of PHDs, resulting in more synthesis of EPO. In fact, Wagner *et al*. also reported that EPO levels were negatively associated with ferritin levels even after adjustment for Hgb levels in diabetic CKD patients^10,17^. According to recent evidence, a negative correlation between EPO and Hgb levels was found only in patients with early CKD and not in those with advanced CKD^18^. In the present study, less than 10% of the patients had advanced CKD (defined as eGFR <30 mL/min/1.73 m^2^). Because we enrolled only anemic patients, the median EPO levels fell in parallel with CKD progress, which was consistent with the previous reports in patients with Hgb <11 g/dL^19^. Some previous studies, though not all, demonstrated that UACR had a significant negative relationship with EPO levels^10,20–22^. As UACR increases, increased urine loss of EPO^23^ and/or decreased EPO production due to tubulointerstitial damage may occur. However, this relationship was not confirmed in our investigation.

To date, previous reports on the prognostic role of EPO levels in renal outcomes have been inconsistent. One small study of 13 diabetic patients described that low EPO levels were associated with rapid renal function decline^24^. By contrast, in the Health, Aging and Body Composition Study consisting of community-dwelling older adults aged 70–79 years, there was no association between baseline EPO levels and the renal outcome of ≥30% decline in eGFR^25^. Moreover, another study reported that EPO levels did not predict ESKD or the doubling of serum creatinine in diabetic patients with CKD^26^. Differences in the study populations may explain these discordant results. In the Health, Aging and Body Composition Study, approximately 15% and 20% of participants had anemia and diabetes, respectively, whereas only 40% of participants had anemia in the second study. An important observation of our study was that the relationship between low EPO levels and renal deterioration was remarkable in the group with ID. Compared with previous studies, our population consisted of exclusively diabetic patients with anemia or poor iron status^26^, both of which excessively stimulate EPO synthesis. As a result, our unique population clearly revealed the association between EPO levels and the renal outcome of interest.

It is unclear whether low EPO levels are merely concomitant or play a functional role in the progression of renal dysfunction. In diseased kidneys, REPs convert into myofibroblasts and EPO production is disabled, contributing to subsequent renal fibrosis^27^. In the current study, low EPO levels in diabetic patients with anemia predicted rapid eGFR decline, independently of u-LFABP, a tubular biomarker that is known to predict DKD progression^28,29^. This suggests that low EPO levels in the context of anemia/ID are a proxy of tubulointerstitial damage, which is reported to be a predictor of ESKD in type 2 diabetic patients^30^. In other words, appropriate EPO production in the context of anemia/ID reflects the endocrinological reservoir of the kidney. EPO has hemopoietic and extra-hemopoietic functions such as cytoprotection, inhibition of apoptosis, anti-oxidation, and anti-inflammation^31^. An animal study demonstrated that EPO ameliorates renal fibrosis and podocyte injury^32–35^. Furthermore, several observational and randomized controlled studies have suggested that EPO administration may delay the onset of dialysis in predialysis patients^34–36^. Thus, a low EPO concentration may impact the progression of DKD.

Recently, clinical trials revealed that HIF stabilizers increase EPO levels even in dialysis patients^37^. In addition, a few papers demonstrated that sodium-glucose transporter (SGLT) 2 inhibitors, a new class of diabetes medication, elevate EPO levels^38,39^. One possible explanation for the increased EPO levels is that SGLT inhibitor may exacerbate hypoxia in the renal medulla by activating sodium reuptake at the thick ascending limb, leading to improved oxygen supply-demand balance in the renal cortex^40^. Whether long-term renoprotection by HIF stabilizers or SGLT2 inhibitors, if any, can be attributed to HIF activation and resultant EPO synthesis remains to be elucidated.

A strength of the current study was the robustness and significance of the EPO results even after extensive adjustment. We adjusted not only for classical risk factors including baseline renal function, UACR, and Hgb, but also for FGF23 and 25D, both of which have been reported to be independent risk factors for the progression of renal disease^41–43^. We additionally adjusted for u-LFABP, which predicts DKD progression^28,29^. Our study revealed that EPO, which has been spotlighted for decades, has the potential to be a predictive biomarker for renal outcomes, independent of novel markers such as FGF23, 25D, and u-LFABP.

This study has several limitations. First, we cannot extrapolate our findings to non-diabetic or non-anemic populations. Second, the observational nature of the current study precluded establishing a causal relationship between EPO and the renal outcome. Third, the results may have been biased by other unmeasured confounders. Fourth, eGFR slope is a “soft” renal outcome; therefore, further investigation is warranted to evaluate whether low EPO levels predict “hard” renal outcomes, e.g., initiation of renal replacement therapy. Fifth, EPO levels were measured at a single time point. Lastly, the true and/or concomitant etiology of anemia was unclear because laboratory data regarding anemia-related factors, e.g., vitamin B12 and folate, were unavailable.

In conclusion, low EPO levels, which are common even in anemic patients without CKD, predict rapid renal decline in type 2 diabetic patients with anemia. Our results should encourage clinicians to assess EPO levels in diabetic patients with anemia. Further research is needed to determine whether^1^ low EPO levels predict hard renal outcomes^2^, low EPO levels predict renal outcomes in diverse populations, and^3^ administration of a HIF stabilizer improves renal outcomes in diabetic patients with anemia by elevating serum EPO levels.

## References

[1] Mehdi U, Toto RD (2009). Anemia, diabetes, and chronic kidney disease. Diabetes care.

[2] Heerspink HJL (2010). Comparison of different measures of urinary protein excretion for prediction of renal events. J Am Soc Nephrol.

[3] Drury PL (2011). Estimated glomerular filtration rate and albuminuria are independent predictors of cardiovascular events and death in type 2 diabetes mellitus: the Fenofibrate Intervention and Event Lowering in Diabetes (FIELD) study. Diabetologia.

[4] Porrini E (2015). Non-proteinuric pathways in loss of renal function in patients with type 2 diabetes. *The lancet*. Diabetes & endocrinology.

[5] Sahay M (2017). Diabetes and Anemia: International Diabetes Federation (IDF) - Southeast Asian Region (SEAR) position statement. Diabetes & metabolic syndrome.

[6] Mohanram A (2004). Anemia and end-stage renal disease in patients with type 2 diabetes and nephropathy. Kidney international.

[7] Ueda H (2003). Factors affecting progression of renal failure in patients with type 2 diabetes. Diabetes care.

[8] Babazono T (2006). Lower haemoglobin level and subsequent decline in kidney function in type 2 diabetic adults without clinical albuminuria. Diabetologia.

[9] Belonje AM (2010). Endogenous erythropoietin and outcome in heart failure. Circulation.

[10] Wagner M (2011). Endogenous erythropoietin and the association with inflammation and mortality in diabetic chronic kidney disease. Clinical journal of the American Society of Nephrology: CJASN.

[11] Molnar MZ (2011). Serum erythropoietin level and mortality in kidney transplant recipients. Clinical journal of the American Society of Nephrology: CJASN.

[12] Ichihara K (2012). Setting of the joint ownership standard range by the central measurement system. Japanese Journal of Clinical Laboratory Automation.

[13] Matsuo S (2009). Revised equations for estimated GFR from serum creatinine in Japan. American journal of kidney diseases: the official journal of the National Kidney Foundation.

[14] Yamamoto H (2017). 2015 Japanese Society for Dialysis Therapy: Guidelines for Renal Anemia in Chronic Kidney Disease. Renal Replacement Therapy.

[15] Symeonidis A (2006). Inappropriately low erythropoietin response for the degree of anemia in patients with noninsulin-dependent diabetes mellitus. Annals of hematology.

[16] Bosman DR, Winkler AS, Marsden JT, Macdougall IC, Watkins PJ (2001). Anemia with erythropoietin deficiency occurs early in diabetic nephropathy. Diabetes care.

[17] Shah YM, Xie L (2014). Hypoxia-inducible factors link iron homeostasis and erythropoiesis. Gastroenterology.

[18] Mercadal L (2012). Timing and determinants of erythropoietin deficiency in chronic kidney disease. Clinical journal of the American Society of Nephrology: CJASN.

[19] Fehr T (2004). Interpretation of erythropoietin levels in patients with various degrees of renal insufficiency and anemia. Kidney international.

[20] Inoue A, Babazono T, Suzuki K, Iwamoto Y (2007). Albuminuria is an independent predictor of decreased serum erythropoietin levels in type 2 diabetic patients. Nephrology, dialysis, transplantation: official publication of the European Dialysis and Transplant Association - European Renal Association.

[21] Mojiminiyi OA (2006). Prevalence and associations of low plasma erythropoietin in patients with Type 2 diabetes mellitus. Diabetic medicine: a journal of the British Diabetic Association.

[22] Nagai T (2016). Prognostic significance of endogenous erythropoietin in long-term outcome of patients with acute decompensated heart failure. European journal of heart failure.

[23] Vaziri ND, Kaupke CJ, Barton CH, Gonzales E (1992). Plasma concentration and urinary excretion of erythropoietin in adult nephrotic syndrome. The American Journal of Medicine.

[24] Inomata S, Itoh M, Imai H, Sato T (1997). Serum levels of erythropoietin as a novel marker reflecting the severity of diabetic nephropathy. Nephron.

[25] Garimella PS (2016). Association of Serum Erythropoietin With Cardiovascular Events, Kidney Function Decline, and Mortality: The Health Aging and Body Composition Study. Circulation. Heart failure.

[26] Wagner M (2015). Hepcidin-25 in diabetic chronic kidney disease is predictive for mortality and progression to end stage renal disease. PloS one.

[27] Asada N (2011). Dysfunction of fibroblasts of extrarenal origin underlies renal fibrosis and renal anemia in mice. The Journal of clinical investigation.

[28] Kamijo-Ikemori A (2011). Clinical significance of urinary liver-type fatty acid-binding protein in diabetic nephropathy of type 2 diabetic patients. Diabetes care.

[29] Nielsen SE (2010). Urinary liver-type fatty acid-binding protein predicts progression to nephropathy in type 1 diabetic patients. Diabetes care.

[30] Mise K (2016). Prognostic Value of Tubulointerstitial Lesions, Urinary N-Acetyl-beta-d-Glucosaminidase, and Urinary beta2-Microglobulin in Patients with Type 2 Diabetes and Biopsy-Proven Diabetic Nephropathy. Clinical journal of the American Society of Nephrology: CJASN.

[31] Singh DK, Winocour P, Farrington K (2009). Erythropoietic stress and anemia in diabetes mellitus. Nature reviews. Endocrinology.

[32] Loeffler I, Rüster C, Franke S, Liebisch M, Wolf G (2013). Erythropoietin ameliorates podocyte injury in advanced diabetic nephropathy in the db/db mouse. American Journal of Physiology-Renal Physiology.

[33] Park SH (2007). Erythropoietin decreases renal fibrosis in mice with ureteral obstruction: role of inhibiting TGF-beta-induced epithelial-to-mesenchymal transition. J Am Soc Nephrol.

[34] Kuriyama S (1997). Reversal of anemia by erythropoietin therapy retards the progression of chronic renal failure, especially in nondiabetic patients. Nephron.

[35] Jungers P (2001). Beneficial influence of recombinant human erythropoietin therapy on the rate of progression of chronic renal failure in predialysis patients. Nephrology, dialysis, transplantation: official publication of the European Dialysis and Transplant Association - European Renal Association.

[36] Gouva C, Nikolopoulos P, Ioannidis JP, Siamopoulos KC (2004). Treating anemia early in renal failure patients slows the decline of renal function: a randomized controlled trial. Kidney international.

[37] Brigandi RA (2016). A Novel Hypoxia-Inducible Factor-Prolyl Hydroxylase Inhibitor (GSK1278863) for Anemia in CKD: A 28-Day, Phase 2A Randomized Trial. American journal of kidney diseases: the official journal of the National Kidney Foundation.

[38] Heerspink HJL, de Zeeuw D, Wie L, Leslie B, List J (2013). Dapagliflozin a glucose-regulating drug with diuretic properties in subjects with type 2 diabetes. Diabetes, obesity & metabolism.

[39] Ferrannini E (2017). Renal Handling of Ketones in Response to Sodium-Glucose Cotransporter 2 Inhibition in Patients With Type 2 Diabetes. Diabetes care.

[40] O’Neill J (2015). Acute SGLT inhibition normalizes O2 tension in the renal cortex but causes hypoxia in the renal medulla in anaesthetized control and diabetic rats. American journal of physiology. Renal physiology.

[41] Nakano C (2012). Combined use of vitamin D status and FGF23 for risk stratification of renal outcome. Clinical journal of the American Society of Nephrology: CJASN.

[42] Hamano T (2013). Fibroblast growth factor 23 and 25-hydroxyvitamin D levels are associated with estimated glomerular filtration rate decline. Kidney international supplements.

[43] Titan SM (2011). FGF-23 as a predictor of renal outcome in diabetic nephropathy. Clinical journal of the American Society of Nephrology: CJASN.

